# Enhancing the Delivery of Resveratrol in Humans: If Low Bioavailability is the Problem, What is the Solution?

**DOI:** 10.3390/molecules191117154

**Published:** 2014-10-24

**Authors:** James M. Smoliga, Otis Blanchard

**Affiliations:** 1 Department of Physical Therapy, School of Health Sciences, High Point University, High Point, NC 27262, USA; 2 Department of Basic Pharmaceutical Sciences, School of Pharmacy, High Point University, High Point, NC 27262, USA; 3 Wilmore Labs LLC, San Antonio, TX 78250, USA

**Keywords:** pharmacokinetics, pharmacodynamics, phytochemical, nutraceutical, red wine, sirtuins, SIRT1, stilbene

## Abstract

Resveratrol has emerged as a leading candidate for improving healthspan through potentially slowing the aging process and preventing chronic diseases. The poor bioavailability of resveratrol in humans has been a major concern for translating basic science findings into clinical utility. Although a number of positive findings have emerged from human clinical trials, there remain many conflicting results, which may partially be attributed to the dosing protocols used. A number of theoretical solutions have been developed to improve the bioavailability of resveratrol, including consumption with various foods, micronized powders, combining it with additional phytochemicals, controlled release devices, and nanotechnological formulations. While laboratory models indicate these approaches all have potential to improve bioavailability of resveratrol and optimize its clinical utility, there is surprisingly very little data regarding the bioavailability of resveratrol in humans. If bioavailability is indeed a limitation in the clinical utility of resveratrol, there is a need to further explore methods to optimize bioavailability in humans. This review summarizes the current bioavailability data, focusing on data from humans, and provides suggested directions for future research in this realm.

## 1. Introduction

In recent years, resveratrol has gained notoriety as a front runner in the field of anti-aging [1], which is a perilous title when the mechanism of aging itself is poorly defined [2,3,4,5]. Discussed in the media as “The Red Wine Molecule” beginning around 2003, resveratrol gained fame after it was demonstrated to improve longevity in obese male C57BL/6Nia mice [6,7]. After over a decade of high impact and highly publicized *in vivo* research [8,9,10], this small molecule and the research surrounding it maintains a controversial enigmatic status as its potential clinical utility remains uncertain [11]. The current evidence supports that resveratrol primarily acts via direct and indirect activation of the histone deacetylase SIRT1 in laboratory models [12,13]. SIRT1 activation, governs a complex array of signaling cascades [14,15,16], pertaining to apoptosis, autophagy, and insulin sensitivity and appears to be a major mechanistic component for the benefits of caloric restriction [17,18]. Early research which evaluated the bioavailability of resveratrol in humans suggested that its bioavailability was rather limited [9,19,20]. Though cell culture models using high concentrations of resveratrol, often 10 μM–50 μM or greater, have demonstrated much potential [21], there has been substantial concern that the concentrations used *in vitro* and in animal models are not reasonably attainable *in vivo* [1]. Early research which evaluated the bioavailability of resveratrol in humans suggests that its bioavailability is rather limited [9,19,20]. 

The hope and hype surrounding resveratrol was sufficient to lead to the development of human clinical trials in the absence of a full understanding of its mechanism of action or optimal dosage protocols to attain sufficient concentrations in humans. Human clinical trials exploring the health effects of resveratrol continue to emerge, but remain somewhat limited in scope and number. Much of the human literature has yielded promising results consistent with data from laboratory animal models [22], such that resveratrol reduces biomarkers of inflammation in healthy individuals [23,24,25], improves clinical biomarkers in diabetes [26,27], increases blood flow to the brain [28], and enhances vascular function in general [29,30]. However, conflicting findings between human and animal also have arisen, which have fueled healthy skepticism regarding resveratrol’s ultimate clinical potential. Resolutions for such conflicts are made difficult with the observation that the beneficial effects have been attained using dosages far below those required in cell culture [11,21,31]. In the simplest sense, the contradiction between resveratrol’s physiological effects in model organisms and humans may be due to differences in dosing protocols and inappropriate statistical and research methodologies [11,31,32,33]. 

While many human clinical trial results are promising, there are many challenges to overcome in developing resveratrol into effective therapeutic agents [34]. From the most consistent data presented in the literature, one of the major issues surrounding resveratrol’s future may be achieving adequate bioavailability at tolerable doses—a common issue in translating promising findings from cell culture and animal models into clinical efficacious drugs. There are a number of mechanisms to explore which can be employed to potentially enhance the delivery of resveratrol to achieve therapeutic range. Despite a wealth of animal data, only a limited number of these have been attempted in humans. As such, the purpose of this review is to briefly describe the approaches that are being explored to improve the bioavailability of resveratrol with a focus on results from human trials exploring resveratrol administration. 

## 2. Pharmacological Considerations

The basic parameters associated with drug activity are maximal plasma concentration (*C*_max_*)*, time to *C*_max_ (t_max_), half-life (t_1/2_), and exposure measured by area under curve (AUC). There is robust evidence that the *in vitro* and *in vivo* response to resveratrol is dose-dependent [35,36,37,38] and possibly exposure-dependent [39]. It is also currently unknown whether maximizing the *C*_max_ or the AUC is most important for the clinical efficacy of resveratrol. For example, there is evidence that even two minutes of exposure to resveratrol has beneficial effects on nitric oxide production for *in vitro* models [40]. Regardless of which type of exposure is most important, sufficient quantities of resveratrol must first be absorbed into the bloodstream and be delivered to target tissues. 

Resveratrol is absorbed at a relatively high rate through the small intestine [41]. The small and non-polar character of *trans*-resveratrol may allow for its absorption across the membranes by passive diffusion [41], yet there is compelling evidence that resveratrol is chiefly transported across the intestinal epithelium via cell via ATP-dependent binding cassette (ABC) transporters [42]. Inside the enterocytes of the small intestine and hepatocytes of the liver, the glucuronide and sulfate conjugation of *trans*-resveratrol to the major metabolites is extensive [9,43,44,45]. This conjugation to sulfates and glucuronides increases resveratrol’s aqueous solubility, and reduces flux across membranes preventing non-polar molecules from interacting with essential macromolecules and allows for excretion by the kidneys via urine [9,45]. The extensive metabolism to glucuronide and sulfate conjugates during absorption is well described, and decreases circulating levels of free *trans-*resveratrol. Thus, metabolism of resveratrol ultimately results in relatively small amounts of free *trans*-resveratrol in the plasma to be delivered to other tissues. Additionally, there is considerable inter-individual variability in bioavailability of resveratrol [9,20], which may cause inconsistent physiologic responses between individuals and limit clinical applicability [11]. 

Human clinical trials have focused on single or multiple oral dosages of resveratrol capsules and tablets, and the majority of bioavailability data in humans reflect this delivery method. These oral dosages have been administered through various dose regimens, sizes, and physiological formulations [27,45,46,47]. A number of factors ultimately influence the pharmacokinetics parameters of oral delivery, including factors related to the molecule itself, physiology, and individual characteristics [11,48,49]. For the small molecule itself, this may include the size of the molecule, charge at physiological pH, solubility, rate of active and passive absorption in the gastrointestinal tract, and the enzyme activity of the first pass metabolism [42,43,44]. Further, the photostability of the resveratrol itself must also be considered when developing formulations [50]. Strategies to increase bioavailability from oral delivery of resveratrol are generally focused on increasing the rate of resveratrol absorption into the enterocytes and decreasing intracellular metabolism [46,51,52,53]. Many of these strategies that are in early stages of development are described in detail by Amri *et al.* [53].

## 3. Bioavailability in Humans Following Standard Oral Dosing

Table 1 displays a summary of key bioavailability parameters from published human studies and offers comparisons regarding the pharmacokinetics of resveratrol between human studies. The greatest *C*_max_ and AUC of free *trans*-resveratrol have been observed following the largest dosages, generally 5 g per day [23,45,54]. These doses demonstrate that a *C*_max_ of approximately 4 µM is attainable in human plasma, and this may be sufficient to achieve some of the physiological benefits demonstrated in laboratory models from *trans*-resveratrol alone [35,45,55]. Theoretically, greater dosages could be used to maximize resveratrol exposure, but larger dosages of resveratrol may also be associated with poor tolerance [23,54]. Thus, considerable effort has been put forth to increase the ratio between free *trans*-resveratrol concentration and dosage administered. 

One simple approach to enhance bioavailability has been combining various food and beverage consumption with resveratrol administration, with the notion that the combination of resveratrol with multiple polyphenols is ultimately responsible for the “French Paradox”. Goldberg *et al.* [56] found similar plasma AUC for total resveratrol (parent compound combined with metabolites) following administration of 25 mg resveratrol with grape juice, V8 juice, or red wine, though resveratrol clearance was slowest following grape juice. Despite much interest on the synergistic effects of resveratrol with red wine polyphenols, no further published works have explored the combination of resveratrol supplementation with other beverages. There is only one study exploring the influence of food intake on resveratrol, whereby LaPorte *et al.* [57] reported a standard breakfast with 2000 mg resveratrol supplementation yielded a significantly greater *C*_max_ and AUC than that obtained following a high fat breakfast. Unfortunately, neither of these studies included a control condition to determine whether food or beverages enhanced or impaired bioavailability compared to resveratrol itself. However, Vas da Silva *et al.* [58] found no major differences in bioavailability parameters between fed and fasted conditions following 400 mg of resveratrol administration, with the exception of a longer time to maximum plasma concentration. Beyond these studies, there is little known regarding how diet influences bioavailability of resveratrol supplements. 

Another approach to increase the absorption of resveratrol in the gastrointestinal tract is improving the material properties of resveratrol used in the oral dosage. This is the basis for SRT501, the patented formulation of resveratrol which is micronized with particle sizes < 5 µm and solubilized. As described in Howells *et al.*, a finished liquid oral formulation, it is formed as five grams of micronized resveratrol, four milliliter mixture of flavorings, colorings, and emulsifiers such as dousate sodium brought up to twenty milliliters of water for ingestion[54]. The small particle size with the emulsifiers in solution theoretically increases surface area for intestinal absorption while also improving suspension properties [54]. When five grams of resveratrol are administered in SRT501 formulation there is nearly a fourfold increase in peak plasma level, from 538 ng/mL (2.36 µM) to 1942 ng/mL (8.51 µM) and the time to maximum plasma concentration nearly doubled from 1.5 h to 2.8 h [45,54]. The comparison of the 5 g single dosage on AUC increased from 1319 ng h/mL to 6327 ng h/mL at this highest reported dosage. 

Increases in solubility, such as that of SRT501, are widely known to influence absorption from increasing the amount of drug in free form, but there may be a limit to this effect. Das *et al.* [51] improved aqueous solubility nearly 60,000 fold using hydroxypropyl-β-cyclodextrin, and this formulation, which increases rate of absorption, had a significant impact on the maximal plasma concentration, yet negligible impact on the bioavailability of resveratrol in rats. One can also consider the possibility that the binding of resveratrol to the hydrophobic center cyclodextrin may be similar to resveratrol self-association in the gastrointestinal tract affecting the extent of absorption, such that dissociation and consequent absorption is limited [48]. The contrasting results between SRT501 and cyclodextrin raise the possibility that there is an optimal solubility for gastrointestinal absorption, or that this is a specific issue in rodent models. Further research into this is necessary

## 4. Synergetic/Additive Interactions

A variety of other molecules have been demonstrated to have synergistic and additive effects when combined with resveratrol for *in vitro* models, and such interactions may potentially enhance bioavailability. Protecting resveratrol from rapid metabolization in the gastrointestinal tract and liver is one general mechanism which can increase bioavailability. Given that CYP, UGT, and SULT are the key enzymes which conjugate resveratrol, any intervention which decreases their rate of reaction on *trans*-resveratrol should increase concentration of the parent compound. For this reason, it has been suggested that combining resveratrol with other polyphenols which are targeted by these enzymes may increase bioavailability. 

Piperine, a polyphenol found in black pepper, has been shown to substantially increase serum *C*_max_ and AUC of resveratrol in rats [59]. In coadministration in an oral gavage of 100 mg/kg and 10 mg/kg piperine, Johnson *et al.* showed a 1000% increase of peak plasma levels for resveratrol while delaying a major glucuronide resveratrol metabolite [59]. Consequent research in cellular [60] and animal [61] models has indeed shown piperine to potentiate the effects of resveratrol. However, only one human study has explored this combination, and found that piperine may enhance resveratrol’s effects on cerebral blood flow, yet does not increase its bioavailability [62]. The failure to increase bioavailability may be due to a lower mg/kg dosage in humans for piperine and resveratrol, which prevented a saturation of the UGT enzyme and had no effect on the rate of metabolite formation. Alternatively, this may be a difference in human and rat UGT1A1 isoforms, where piperine may not inhibit UGT in humans as has been observed in rodent models [63]. 

Similarly, attempts to increase the bioavailability of resveratrol through combining it with quercetin to inhibit SULT1A1 [64]. In an *ex vivo* kinetics study with cytosolic fraction of homogenized rat liver, the rate of resveratrol sulfation was reduced with the addition of purified quercetin [65]. Quercetin may reduce the rate of resveratrol sulfate formation, but this does not *necessarily* equate to improved bioavailability for resveratrol. *In vivo*, LaPorte found that adding 500 mg of quercetin to 2000 mg of resveratrol did not enhance the *C*_max_ or AUC, and this was not influenced by the addition of ethanol [57]. This could be due to the concentration of resveratrol or quercetin never reaching the point of saturating and/or inhibiting sulfatransferases *in vivo*, resulting in little reduction in the rate of metabolite formation. Alternatively, the absorption of native quercetin is poor, and may not reach the liver in a concentration that will inhibit SULT *in vivo* [66].

## 5. Resveratrol Precursors/Pro-Drugs

Another approach to maximize the bioavailability of free *trans*-resveratrol is to develop resveratrol prodrugs. Assuming that maximizing free *trans*-resveratrol is the primary goal, the theoretical ideal is that a resveratrol prodrug would allow resveratrol to be generated through enzymatic reactions *in vivo* to allow for a physiological relevant concentration without toxicity [67]. For a prodrug to be effective, at least one of the following must occur: (1) metabolism of the prodrug to generate high plasma concentrations of resveratrol; (2) entry of the prodrug into tissues of interest, which can then metabolize the prodrug into resveratrol to maximize tissue concentration; (3) acceptable pharmacokinetics of the prodrug itself. 

There has been a single rodent study reporting bioavailability of a resveratrol prodrug, and with promising results. In its formation, the hydrophilic hydroxyl groups or resveratrol are acetylated to 3,5,4'-tri-*O*-acetylresveratrol (taRES) occupying the major sites of glucuronidation and sulfation. This should allow high levels of the prodrug to be maintained, until it is deacetylated to form resveratrol. Liang *et al.* [68] demonstrated intragastric administration of 155 mg/kg taRES to rats resulted in a greater AUC of resveratrol than an equivalent dosage (100 mg/kg) of resveratrol. The increased AUC was likely related to slower elimination, given that *C*_max_ of resveratrol was greater than taRES. Additionally, the vehicle used for taRES can also dramatically extend its half-life [69]. However, taRES may have also biologic activity of its own [70]. Regardless, the prodrug approach has not yet been attempted in humans.

## 6. Alternative to Standard Oral Dosages 

As previously mentioned, oral delivery of resveratrol has received the majority of clinical attention, but it is unclear if the many reported potential micronized and encapsulated formulations will be able to overcome issues faced by the widely explored micronized resveratrol formulation SRT501 [27,53,54,71]. With this in mind, few trials have investigated potential delivery routes beyond traditional oral administration. These methods bypass the constraints to concentration of the gastrointestinal tract and first pass hepatic metabolism, but have their own challenges to overcome. Perhaps the most obvious way to maximize absorption of resveratrol is to bypass the gastrointestinal tract completely and deliver resveratrol directly into the bloodstream. Intravenous administration overcomes the mechanical constraints of gastrointestinal absorption (*i.e.*, contact between resveratrol molecules and enterocytes), while initially preserving parent compound through circumventing conjugation by the enterocytes and gut microbiota. Intravenous administration of resveratrol in rats has been demonstrated to result in far greater AUC for resveratrol and selected metabolites compared to oral administration, this effect being magnified at higher intravenous dosages [72].

While intravenous injection may provide greater plasma concentration of free *trans*-resveratrol than typical oral administration, it is not clinically practical if chronic self-administration is desired. However, intravenous administration may be useful to rapidly deliver a large bolus of resveratrol. In rats, resveratrol metabolites are detectable within one minute after intravenous injection via the tail vein, and glucuronide conjugates achieve a greater concentration than parent compound within three minutes [73]. This rapid delivery may produce quick physiological responses which can lead to valuable clinical applications. Indeed, intravenous resveratrol administration has been demonstrated to acutely increase renal blood flow in rats [74] and acutely influence outcomes in various animal models of ischemia [75,76]. Although intravenous administration of resveratrol in animal models has demonstrated great potential, there are no reports of intravenous administration in humans throughout the scientific literature. Nonetheless, there is no known reason for intravenous administration of resveratrol to be contraindicated in humans, and this is an area for further exploration. 

A non-invasive means of resveratrol delivery, which may circumvent the constraints of the gastrointestinal tract and first pass hepatic metabolism, is oral transmucosal dosing [53,77,78]. There are multiple known limitations for the method, such as a limited dose size and difficulties achieving absorption [79,80,81]. Given that free *trans*-resveratrol can travel through the systemic circulation before being metabolized in the liver, tissue likely has greater exposure to free *trans*-resveratrol when it is absorbed through the oral mucosa. Blanchard *et al.* [78] has reported a successful proof of concept study where a *C*_max_ of ~1.4 µM in two healthy humans following administration of a 140 mg buccal dosage of resveratrol. This is considerably greater than that reported for a standard oral dosage [47], however a direct comparison was not performed. Current concerns regarding oral transmucosal deliver center on the rapid decrease in parent compound from the plasma. Similar to Yeo *et al.*, who reports very rapid time to *C*_max_ using a sublingual dosage of pterostilbene in anesthetized rats, Blanchard *et al.* reports a high plasma concentration 15 min post-administration, but no measureable free *trans*-resveratrol within 30 min post-administration [78,82]. This is similar to what has been previously reported with an injected dosage of resveratrol, where rapid clearance is a result of distribution to tissues and the phase II metabolism to sulfate conjugates [9,83]. While human data demonstrates promise in oral transmucosal delivery, further work is needed in this realm.

One potential method to maximize free *trans*-resveratrol concentration near the tissue of interest is through implantable devices, and this approach has been demonstrated to be promising in animal models. While currently approved drug eluting stents have made major improvements in restenosis over bare metal stents, they have also been shown to prevent vascular wound healing [84]. This prevention of healing is understood to be a factor in developing late thrombosis with Taxol and rapamycin derivative stents [84,85,86]. In 2010, Khanderwal *et al.* showed that orally delivered resveratrol inhibited stenosis in a dose-dependent fashion via ER-alpha [87] and then developed stents with resveratrol as a primary active ingredient. While these stents with resveratrol were not directly compared to the current drug eluting stents, the authors report a comparable impact over bare stents [85]. Recently the group showed that resveratrol promotes wound closure compared to Taxol *in vitro* [86], which shows promise for further application *in vivo*. While this application likely increases local bioavailability, its systemic effects remain unknown, and these have not yet been tested in humans. Nonetheless, such localized delivery systems hold potential for clinical utility.

## 7. Nanotechnological Approaches

A number of recent studies have focused on using nanotechnology to improve the bioavailability of resveratrol and have generally demonstrated improved stability and bioavailability with minimal side effects compared to oral dosing. Nanoformulations can improve resveratrol’s solubility and transport across the plasma membrane, and thus enhance its effects within cells [77]. There is emerging evidence that nanoformulations of resveratrol can protect resveratrol from metabolism during the digestive process, which ultimately increases tissue absorption in animal models [88]. Resveratrol loaded onto lipid-core nanocapsules improved tissue concentration in the brain, liver, and kidney of healthy rats compared to free resveratrol [89]. Additionally, the nanocapsule formulation decreased gastrointestinal damage in rats [89], which suggests such a formulation could improve tolerability in humans while also increasing bioavailability [89]. In an ovarian cancer model, resveratrol-bovine serum albumin nanoparticles initially demonstrated greater blood concentration than resveratrol alone following intraperitoneal injection [90]. However, the nanoparticle formulation achieved greater tissue distribution to the liver, heart, kidney, and ovary, and ultimately was more effective than standard resveratrol [90]. Likewise, resveratrol loaded solid nanoparticles demonstrate selectively greater distribution to the brain, yet similar or even lower distribution to other tissues compared to free resveratrol [52]. This is especially important, given that resveratrol distribution to the brain is normally very limited compared to that in other tissues [91,92,93]. Thorough reviews of nanotechnological approaches to enhance the bioavailability of resveratrol and other phytochemicals have recently been published [94,95,96]. Though nanotechnology holds much promise for enhancing the bioavailability of resveratrol, considerable work must be done in this realm, which may be specific to intended use (e.g., general cardiovascular *vs.* cancer chemotherapeutic) and route of delivery (e.g., dermal *vs.* oral *vs.* intravenous). Nonetheless, these nanotechnological approaches are yet to be attempted in humans.

## 8. Metabolite Activity-Implications for Bioavailability

Controversy remains as to whether resveratrol itself is the primary molecule responsible for health benefits, or whether the activity of its metabolites contributes significantly to its biological effects [97,98]. If resveratrol metabolites have a similar or greater magnitude of physiologic activity and tissue distribution compared to resveratrol itself, this could lead to a paradigm shift and dramatically change the future direction of dosing research, with less emphasis needed on parent compound. It has also been suggested that resveratrol metabolites may serve as a reserve for parent compound to be regenerated, thus serving as prodrugs, and this has been referred to as “recycling” [99]. 

There is evidence that at sufficient concentrations of resveratrol metabolites have biologic activity in various tissues, and a thorough description of metabolite activity is available elsewhere [67]. The uncertainty of this issue is well exemplified in examining the response of adipose tissue to resveratrol and its metabolites *in vitro*. For instance, 25 μM resveratrol and its glucuronide and sulfate metabolites decreased triacylglycerol content in maturing pre-adipocytes, though only parent compound and glucuronide metabolite did so in mature adipocytes [100]. Interestingly, the delipidating response was triggered at lower concentrations of parent compound (1 μM) than glucuronide metabolites (10 μM). Generally, resveratrol itself triggered greater responses in mRNA expression of various biomarkers, though one specific glucuronide metabolite influenced selected markers of lipolysis [100,101]. There is some evidence that resveratrol itself has greater activity than selected metabolites on various targets, such as SIRT1, COX1, and COX2, though the magnitude of the difference between compounds varies and their clinical relevance remains currently unknown [98,102]. However, metabolite activity is not universal across targets, as there is also evidence that resveratrol metabolites have no effects in some tissues where resveratrol itself is effective [55]. Further, it is not yet known if the metabolites can act as resveratrol prodrugs in all tissues, as enzymes involved in recycling (e.g., SULT1A1 [44]) are not expressed universally [103]. Taken together, this data suggest that the metabolites of resveratrol do have biologic significance, either through direct activity or recycling, and is not yet sufficient information to determine the relevance of metabolites *in vivo*. 

Evidence of metabolite activity and recycling in certain tissues necessitates some consideration of their bioavailability, but the bioavailability of resveratrol itself currently remains most relevant. When measuring pharmacokinetics and activity from a prodrug it is customary to focus on the levels of the active molecule [48], and thus the bioavailability of resveratrol itself remains relevant given the current state of the literature. Regardless, metabolites contribute to the majority of total resveratrol concentration following oral administration, and thus there are logically few options to further increase the metabolite levels *in vivo* [9,23,45]. For instance, Boocock [45] reported *C*_max_ for two separate mono-glucuronide metabolites, for a total glucuronide metabolite concentration ~7.5 µM following a single 5.0 g oral dosage of resveratrol. Even at this high dosage, the metabolite concentration remains well below the concentrations used for *in vitro* experiments. Thus, even if metabolites play a role in resveratrol’s *in vivo* physiological effects, strategies to enhance bioavailability remain highly relevant.

## 9. Limitations of Current Resveratrol Bioavailability Research

There are clearly many possible approaches to increase the bioavailability of *trans*-resveratrol, but there remains much uncertainty. While larger doses appear to be associated with greater bioavailability, there is considerable variability in the results between studies. For instance, Boocock *et al.* [45] reports a *C*_max_ =538.8 ng/mL (~2.4 µM) following a single dose of 5000 mg resveratrol, but this is far lower than the 1274 ng/mL (~5.6 µM) reported by LaPorte *et al.* [57] following a much lower dosage (2000 mg with a standard breakfast) [45,57]. Likewise, Amoit *et al.* [46] reports an approximate *C*_max_ >350 ng/mL following a single dose of 40 mg non-optimized resveratrol powder, which is greater than that reported by Almeida *et al.* [47] following 150 mg (24.8 ng/mL), Kennedy *et al.* [28] following 500 mg (14.4 ng/mL, which is lower than Almeida [82], despite a larger dosage), or Boocock *et al.* [45] following 2500 mg (268 ng/mL). The origins of these discrepancies are not definitively known, but may be due to different quantification techniques (e.g., HPLC *vs.* MS/MS, *etc.*) or different formulations of resveratrol. Nonetheless, the wide variation of data between studies makes it incredibly difficult to compare bioavailability of different formulations. If there truly is this much variability in free *trans*-resveratrol bioavailability between different resveratrol products, it should not be surprising that the results from human clinical trials using different dosage protocols sometimes conflict one another.

There remains concerns that the concentrations found *in vivo* in humans are still insufficient to elicit beneficial results. Nonetheless, there is sufficient research to demonstrate that even low concentrations of resveratrol are beneficial. Resveratrol has been shown to increase eNOS activity at from 0.1 µM to 1 µM in a human endothelial cell line after only two minutes of incubation [40]. Another recent *in vitro* study has shown that resveratrol increases activation of AMPK, via SIRT1 [104], in human vascular smooth muscle cells at 3 µM concentration. In addition, a human study has shown dose dependent increases in cerebral blood flow from resveratrol at 5.65 and 14.4 ng/mL (0.025 and 0.061 µM) [28]. This data is supportive of the pharmacology of the aforementioned resveratrol stent [105], and novel dosage forms from the activation of eNOS and upstream effectors relating to cardiovascular disease [88,106].

Bioavailability of resveratrol is generally characterized through quantifying plasma or serum concentrations, but this does not consider resveratrol found in red blood cells [107] or that which has been distributed to other tissues. As such, evaluation of resveratrol in plasma represents only a small fraction of that actually found in the blood [108] and may not be an accurate indicator of resveratrol absorption and exposure. Further, it must be remembered that blood represents only one biological target tissue. While removal via the hepatobiliary and renal systems does account for one aspect of the time-dependent clearance from the blood, resveratrol does accumulate in other tissues where it may have beneficial effects, such as the heart, liver, and skeletal muscle [91,92,93,109]. As previously noted, tissue distribution was increased despite a lower blood concentration four hours following administration of a resveratrol nanoparticle formulation [90]. Thus, bioavailability parameters may be very useful in evaluating endothelial exposure, but may not be representative of widespread tissue exposure. The invasiveness of biopsy makes it quite difficult to measure tissue exposure in humans, though Patel has overcome this barrier through utilizing surgical patients [110]. Likewise, skeletal muscle and adipose biopsy is becoming more common in resveratrol human clinical trials [8,111], and therefore it may be incorporated into bioavailability studies. 

## 10. Conclusions

For over a decade, it has been realized that resveratrol has poor bioavailability in humans. In the time since, a number of human clinical trials have been performed using a wide range of dosage protocols, and conflicting findings have caused considerable controversy over clinical utility. While there are a number of promising methods to increase bioavailability of resveratrol, as demonstrated in animal models, surprisingly very little work has been performed in this regard for humans. This is especially concerning, given that conclusions about resveratrol’s future are being generated from human clinical trials which are currently being performed without a full understanding of the optimal dosage protocols. The ultimate clinical potential of resveratrol cannot be fully realized until proper dosage protocols, which provide optimal bioavailability to ensure sufficient tissue distribution, are established. Until then, poor bioavailability is just one of many factors which may account for discrepancies between laboratory models and human clinical trials. Interpretation of human bioavailability literature is complicated by major differences between dosing protocols and quantification methods, with a lack of comparative bioavailability studies. Lastly, current bioavailability measurements may not fully represent the total resveratrol pool available in the blood and generally do not quantify tissue distribution. Whenever possible, future research exploring the bioavailability of resveratrol in humans should:

(1) Include a control resveratrol condition (e.g., standard oral dosage) that can serve as a reference to determine the effectiveness of novel formulations.
(2) Explore a variety of dosages to determine how bioavailability parameters, including metabolite distribution, are influenced by quantity of resveratrol administered.
(3) Measure concentration of resveratrol and its metabolites in the plasma and whole blood, rather than the plasma alone.
(4) Include tissue samples when ethical and clinically feasible (e.g., bioavailability studies in patients undergoing surgical resection, as part of studies requiring muscle and adipose tissue biopsies, *etc.*).

**Table 1:** Summary of key bioavailability parameters of free *trans*-resveratrol in humans following oral supplement administration.

Author (Year) [Ref]	Dosage (mg)	Days	Times/Day	Subjects (n) [Other Notes]	*C*_max_ (ng/mL) [SD]	t_max_ (h) [range]	t_1/2_ (h) [SD ^1^]	AUC_0-__∞_ (ng h/mL) [SD]
Almeida ^1^ (2009) [47]	25	1	1	Healthy volunteers (8 per dosage group)	1.48 [40.3%]	1.0 [0.3–4.0]	2.0 [104%]	0.814 [55.7%]
	50				6.6 [87.5%]	0.9 [0.3–3.0]	1.8 [149%]	4.27 [65.6%]
	100				21.4 [113%]	1.3 [0.5–3.0]	1.1 [44.8%]	19.5 [86.4%]
	150				24.8 [79.4%]	1.3 [0.5–4.0]	1.9 [72.9%]	32 [61.2%]
	25	13	1		3.89 [66.4%]	1.5 [0.8–3.0]	NR	3.1 [70.8%]
	50				7.39 [62.7%]	0.8 [0.5–3.0]	3.2 [51.0%]	11.2 [69.9%]
	100				23.1 [74.2%]	1.1 [0.3–3.0]	2.4 [42.6%]	33 [60.4%]
	150				63.8 [50.0%]	0.8 [0.5–3.0]	4.8 [78.9%]	78.9 [46.8%]
Boocock ^1^ (2007) [45]	500	1	1	Healthy volunteers (10 per dosage group)	72.6 [48.9]	0.83 [0.5–1.5]	2.85 [NR]	223.7 [NR]
	1000				117 [73.1]	0.76 [0.5–4.0]	8.87 [91.1]	544.8 [57.2]
	2500				268 [55.3]	1.38 [0.5–4.0]	4.22 [51.6]	786.5 [36.2]
	5000				538.8 [72.5]	1.5 [0.67–5.0]	8.52 [95.8]	1319 [59.1]
Brown ^1^ (2010) [23]	500	21–28	1	Healthy volunteers (10 per dosage group)	43.8 [89.4%]	1.0 [0.25–5.0]	4.77 [62.1%]	175 [83.7%]
	1000				141 [68.9%]	1.0 [0.25–1.82]	9.7 [37.5%]	503 [79.3%]
	2500				331 [59.2%]	1.0 [0.23–4.97]	9.17 [42.0%]	1250 [40.0%]
	5000				967 [53.5%]	1.08 [0.5–1.5]	7.85 [25.1%]	4097 [107%]
Howells (2011) [54]	5000	14	1	Colorectal and hepatic cancer patients (9) [SRT501]	1942 [1422]	2.8 [1.1]	1.06 [0.39]	6327 [2247]
Kennedy (2010) [28]	250	1	1	Healthy volunteers (22)	5.65 [NR]	1.5 [NR]	NR	NR
	500				14.4 [NR]	1.5 [NR]	NR	NR
LaPorte (2010) [57]	2000 mg	1	1	Healthy volunteers (8) [+Standard breakfast]	1274 [790]	3 [3–4.5]	2.4 [1.4]	3558 [2195]
				[+500 mg quercetin, standard breakfast"]	1296 [627]	4 [3–4]	2.2 [0.5]	4025 [1745]
				[+500 mg quercetin, standard breakfast wih 5% ethanol]	1272 [613]	3 [2.5–4]	2.1 [0.4]	3800 [1482]
				[+High fat breakfast]	689 [345]	5 [4.5–5]	2.5 [0.8]	1966 [643]
Nunes (2009) [112]	200	1	1	Young Males (6)	23.5 [7.4]	0.8 [0.5–1.5]	3.3 [2.4]	56.1 [35.1]
	200	3	3		31.6 [19.4]	1.5 [0.3–3.0]	4.7 [1.6]	116 [83.4]
	200	1	1	Young Females (6)	26.3 [14.5]	1.1 [0.5–3.0]	3.1 [1.5]	51.2 [27.5]
	200	3	3		30.5 [21.5]	1.1 [0.5–3.0]	3.6 [1.5]	111 [74.3]
	200	1	1	Elderly Males (6)	21.6 [9.7]	0.8 [0.5–3.0]	3.2 [0.9]	58.6 [21.2]
	200	3	3		34.5 [32.1]	1.3 [0.5–3.0]	2.9 [1.6]	76.8 [40.0]
	200	1	1	Elderly Females (6)	28.0 [22.0]	0.6 [0.5–3.0]	2.8 [1.2]	68.9 [37.1]
	200	3	3		27.1 [14.4]	1.0 [0.5–2.0]	2.5 [0.8]	90.9 [44.5]
Vaz-da-Silva (2008) [58]	400 mg	1	1	Healthy volunteers (24) [Fasting condition]	47.3 [63.5%]	0.5 [0.25–4.0]	5.9 [42.3%]	128 [53.6%]
				[Fed condition]	42.2 [86.6%]	2.0 [0.25–16.0]	5.6 [41.4%]	131 [46.7%]

^1^ Standard deviations not provided by authors. Coefficient of variations reported instead. NR = Not reported.
